# Functional Outcomes in Survivors of Pediatric Sepsis: A Scoping Review and Discussion of Implications for Low- and Middle-Income Countries

**DOI:** 10.3389/fped.2022.762179

**Published:** 2022-03-08

**Authors:** Namita Ravikumar, Jhuma Sankar, Rashmi Ranjan Das

**Affiliations:** ^1^Division of Pediatric Pulmonology and Critical Care, Department of Pediatrics, All India Institute of Medical Sciences, New Delhi, India; ^2^Department of Pediatrics, All India Institute of Medical Sciences, Bhubaneswar, India

**Keywords:** quality of life, sepsis, functional outcomes, Pediatric Overall Cerebral Performance (POPC), Pediatric Cerebral Performance Category (PCPC), Pediatric Quality of Life

## Abstract

**Background:**

Pediatric sepsis is an important cause of mortality and morbidity in low- and middle-income countries (LMIC), where there is a huge burden of infectious diseases. Despite shortage of resources, adapting protocol-based care has reduced sepsis-related deaths but survivors of pediatric sepsis are at risk of poor functional outcomes.

**Objectives:**

To perform a scoping review of the literature on functional outcomes of pediatric sepsis survivors after discharge from the intensive care unit (ICU) and discuss the implications for patients in LMICs. The outcomes include prevalence of survival with reduced functional outcomes or quality of life (QoL) and changes over time during follow-up or recovery, and these outcomes were compared with other groups of children.

**Methods:**

We searched major medical electronic databases for relevant literature from January 2005 until November 2021, including Medline (*via* PubMed), Embase, CINAHL, and Google Scholar databases. We included observational studies and follow-up data from clinical trials involving children/adolescents (≤18 years) who were admitted to pediatric intensive care unit (PICU) and got discharged finally. Major focus was on survivors of sepsis in LMIC. We followed PRISMA guidelines for scoping reviews (PRISM-ScR).

**Results:**

We included eight papers reporting data of functional outcomes in 2,915 children (males = 53%, and comorbidity present in 56.6%). All included studies were either a prospective or retrospective cohort study. Studies were classified as Level II evidence. Disabilities affecting physical, cognitive, psychological, and social function were reported in children following discharge. Overall disability reported ranged between 23 and 50% at hospital discharge or 28 days. Residual disability was reported at 1, 3, 6, and 12 months of follow-up with an overall improving trend. Failure to recover from a baseline HRQL on follow-up was seen in one-third of survivors. Organ dysfunction scores such as pSOFA, PeLOD, vasoactive inotrope score, neurological events, immunocompromised status, need for CPR, and ECMO were associated with poor functional outcome.

**Conclusions:**

The research on functional outcomes in pediatric sepsis survivors is scarce in LMIC. Measuring baseline and follow-up functional status, low-cost interventions to improve management of sepsis, and multidisciplinary teams to identify and treat disabilities may improve functional outcomes.

## Introduction

Sepsis is a state of dysregulated host response to infection and a major cause of mortality and morbidity. Although infectious diseases and sepsis-related mortality have decreased globally, the burden remains high in low-middle income countries (LMIC) (1). Pediatric severe sepsis accounts for >8% of critically ill children with in-hospital mortality around 25%. One-third of children develop progressive organ dysfunction, and nearly one in five survivors show new functional disability (2). Underlying malnutrition, chronic illness, and immunosuppressed state further increase the risk of mortality (3). With Surviving Sepsis Campaign (SSC) and advances in critical care, mortality due to sepsis has decreased (4). However, survivors of sepsis are increasing and continue to require long-term care. Patients recovering from sepsis develop 1-2 new functional limitations with 10–40% cognitive dysfunction (5). Children who recover from sepsis also have a chance of reinfection likely due to loss of adaptive immunity and reduction in lymphocytes (6). Sepsis is therefore a life-changing and disability-inducing event. Characteristics associated with complications after hospital discharge include pre-sepsis health status, acute septic episode determinants, and quality of hospital treatment (7).

Childhood infections like pneumonia, diarrhea, and malaria account for the leading causes of mortality in children under 5 years of age globally as per the WHO fact sheet of 2019 (8). Integrated Management of Neonatal and Childhood Illness (IMNCI) programs to assess, manage, and refer sick neonates and children at primary and community healthcare levels have resulted in substantial reduction of mortality (9). The SSC guidelines and American College of Critical Care Medicine (ACCM) Clinical Practice Parameters for Hemodynamic Support of Pediatric and Neonatal Septic Shock provide evidence-based guidelines, sepsis bundles, and checklists to guide management based on the availability of resources (10, 11). Apart from these strategies, antibiotic prophylaxis, immunization, and quality improvement initiatives may reduce the morbidity and mortality by prevention of sepsis (12).

Based on the availability of resources, there is a great disparity in the levels of care provided in different regions around the world (13, 14). Critical care includes treatment of children with a life-threatening illness or injury in its broadest sense, without regard for the location and including pre-hospital and emergency and intensive care (15). This is in comparison to intensive care which is provided in the intensive care unit (ICU) with advanced monitoring necessitating sophisticated equipment. In the LMIC, many hospitals lack designated ICUs, adequate number of trained healthcare staff, and access to necessary medications and equipment (16). The focus in these low-resource settings is on developing low-cost, high-yield interventions and aims for improving short-term outcomes (17). Even within each of the LMICs, links between various levels in the pyramid of healthcare are often missing and vary between rural and urban areas (16). Transport of sick patients, emergency stabilization, and tertiary critical care are often suboptimal or lacking. In such areas with limited access to healthcare, follow-up and post-critical care rehabilitation often take a back seat. Consequently, there is substantially high chance of short-term post-discharge mortality as well as poor long-term outcomes (18). Although long-term outcomes among sepsis survivors in developing countries are hardly reported, follow-up in African children showed a post-discharge mortality of 10% within 2 years of pneumonia and 1.5% within 20 days following malaria (19, 20).

## Objectives

The objectives of this review are to perform a scoping review of the literature on the functional outcomes of pediatric sepsis survivors after discharge from the ICU and discuss the implications for patients in LMICs. The outcomes include prevalence of survival with reduced functional outcomes or quality of life (QoL) and changes over time during follow-up or recovery, and these outcomes are compared with other groups of children (if data are available).

## Methods

This scoping review was conducted to map the existing knowledge on the functional outcomes of pediatric patients admitted to the ICU with sepsis. In general, the aim of a scoping review is to collect, analyze, and present a comprehensive map of existing evidence on an important topic (21). We used frameworks and recommendations while conducting this scoping review (22, 23). The stages were (1) research question identification, (2) searching of databases to get relevant studies, (3) study selection, (4) presentation of the data, (5) reporting of the result, and (6) consultation with stakeholders (optional stage). Therefore, our review team consisted of a pediatric intensivist, a general pediatrician, and an information specialist. The PRISMA Extension for Scoping Reviews (PRISMA-ScR) was followed while preparing and reporting the findings of this review (24).

### Types of Studies

#### Inclusion Criteria

Observational studies that report about functional outcomes or QoL in children at discharge as well as during subsequent follow-ups (at 1, 3, 6, and 12 months) were included. The study should have included only children (>1-month to <18-year-age groups) or have outcomes reported separately for children (if both adults and children are the study population).

#### Exclusion Criteria

Studies reporting only mortality, unpublished data, and those not published in English language were excluded.

### Types of Participants

Children of the >1-month to <18-year age groups and both sexes were included.

### Types of Exposure

The type of exposure in this review includes children diagnosed with sepsis or septic shock and admitted to an ICU or pediatric intensive care unit (PICU). Sepsis, severe sepsis, and septic shock are defined as per published guidelines (25).

### Types of Outcome Measures

The outcomes reported include cognitive, physical, psychosocial, and function deficits in activities of daily living that have been assessed on any standard scale within the post-ICU care syndrome (PICS) framework.

### Search Methodology and Study Selection

We searched major medical electronic databases for relevant literature until November 15, 2021, including Medline (*via* PubMed), Embase, CINAHL, and Cochrane CENTRAL. We included observational studies and follow-up data from clinical trials involving children/adolescents (≤18 years) who were admitted to PICUs and got discharged finally. Major focus was on survivors of sepsis in LMIC. The following search terms were used to search PubMed database: (“functional”[All Fields] OR “functionally”[All Fields] OR “functionals”[All Fields] OR “functioned”[All Fields] OR “functioning”[All Fields] OR “functions”[All Fields] OR “physiology”[MeSH Subheading] OR “physiology”[All Fields] OR “function”[All Fields] OR “physiology”[MeSH Terms]) AND (“outcome”[All Fields] OR “outcomes”[All Fields]) AND (“sepsis”[MeSH Terms] OR “sepsis”[All Fields]) AND (“pediatrics”[All Fields] OR “pediatrics”[MeSH Terms] OR “pediatrics”[All Fields] OR “pediatric”[All Fields] OR “pediatric”[All Fields]) AND (“critical care”[MeSH Terms] OR (“critical”[All Fields] AND “care”[All Fields]) OR “critical care”[All Fields]) AND (“patient s”[All Fields] OR “patients”[MeSH Terms] OR “patients”[All Fields] OR “patient”[All Fields] OR “patients s”[All Fields]). Two reviewers (JS and NR) applied the study selection criteria for titles and abstracts and then full texts. Any conflicts regarding inclusion of articles were discussed with the third author (RRD).

### Data Extraction

Data extraction was done using a data extraction form that was designed and pilot tested *a priori*. Two authors (NR, JS) independently extracted the following information from each study: author, year, location (country), study design, details of participants (age, sex, sample size, disease severity, and comorbidity), exposure/intervention details, outcomes (outcome definition, valid unit of measurement, time points of collection and reporting), loss to follow-up, and miscellaneous data (key conclusions, references to other studies, and additional data required).

### Data Synthesis and Presentation of Results

This was done in the following 3 steps: (a) data analysis, (b) reporting of the findings, and (c) discussion of the implications. A narrative synthesis of data was done to analyze and report the findings.

## Results

**Figure 1:**
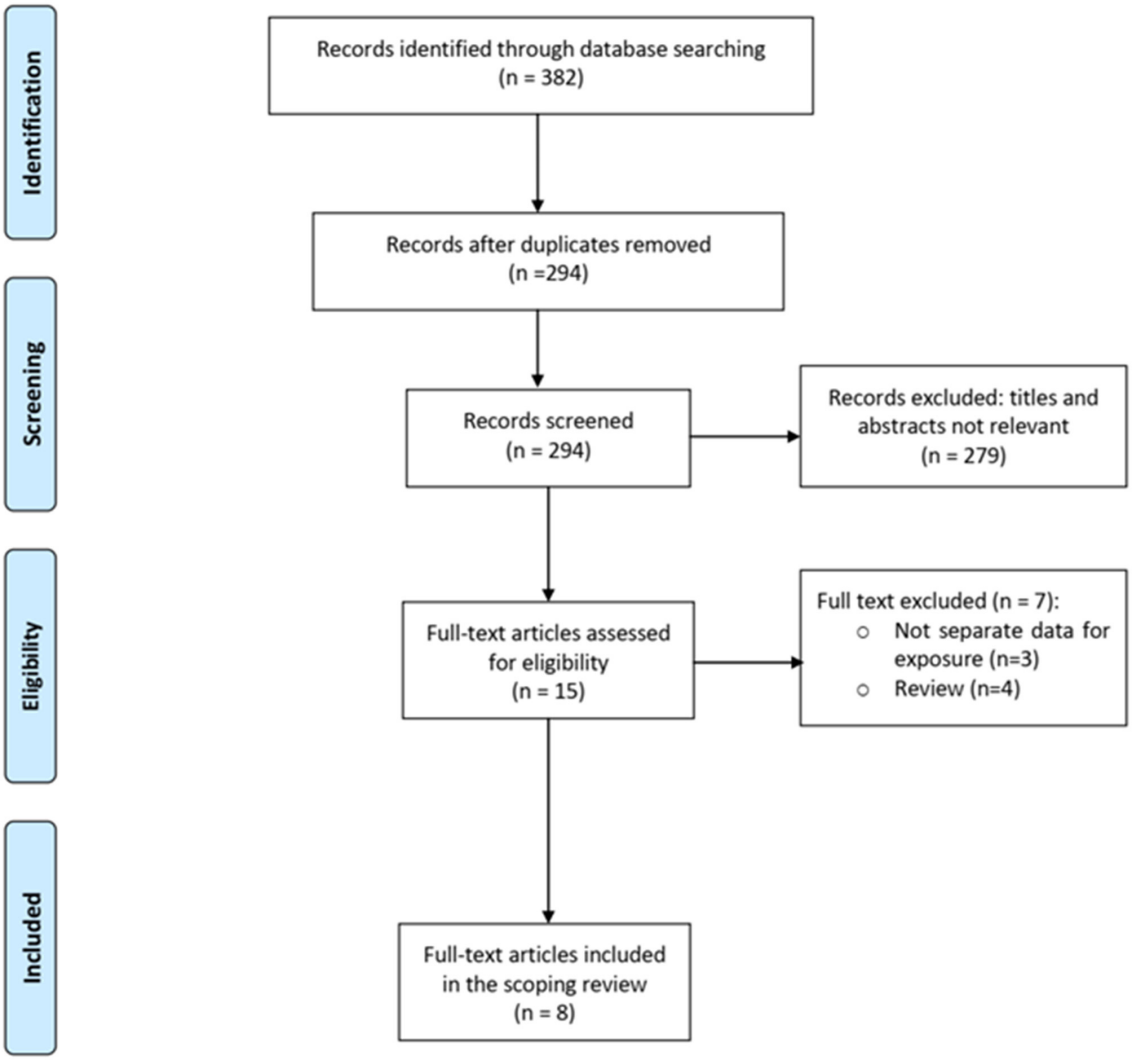
Study flow diagram.

Of 382 total citations retrieved, the full text of 17 papers was assessed for eligibility (Figure 1). Finally, 8 papers including 10,209 children were included (Table 1). Of the 10,209 children, data on functional outcomes were reported for 2,915 children. The age range of the included children was 1 month to 18 years, and 53% were male. Comorbidity was present in 56.6% children. The studies were conducted in the following countries: USA (*n* = 6), Canada (*n* = 1), and India (*n* = 1). Included studies were published from 2013 onward. All studies were cohort studies, both prospective (*n* = 5), and retrospective (*n* = 3). Studies were classified as Level II evidence. Table 1 summarizes the studies on functional outcomes in pediatric sepsis.

**Table 1:** Studies on functional outcomes in pediatric sepsis.

**Study details**	**Study region**	**Patient population**	**Outcome definitions and scores used**	**Results**
Prospective observational study, India—Sankar et al. (26)	LMIC	121 children (2 m-17 y) with severe sepsis	*New disability*—Change in POPC and PCPC ≥1 from baseline *Worse outcomes*—death or new disability at PICU discharge	*Admission*-−33% mild to moderate overall disability and 26% mild to moderate cognitive disability *PICU discharge*-−50.5% new disability in overall function and 28% in cognitive function *At 3 m*—among new disability at PICU discharge improvement in 65% overall and 50% cognitive *At 1 y*—residual disability 5% overall, 14% cognitive PICU mortality 26% Worst outcomes 64% Risk factors—D1 pSOFA and need for CPR
Retrospective cohort study examining data from the RESOLVE trial, multicentric—Farris et al. (27) and Nadel et al. (28)	HIC	477 children (38 wk corrected gestation-17 y) with severe sepsis with cardiovascular and respiratory dysfunction	*Poor functional outcome*—POPC scores 3–5 at day 28 and an increase ≥1 from baseline	34% of survivors had decline in functional status at 28 days and 18% poor functional outcome Risk factors—Hispanic ethnicity, CNS and abdominal infections, recent trauma, CPR prior to enrolment, and baseline PRISM score ≥ 20
Retrospective cohort study, USA—Killien et al. (29)	HIC	790 children (1 m-21 y) with sepsis	*Failure to recover to baseline HRQL*—decrease of ≥4.5 PedsQL from baseline to follow-up	23.8% failed to recover to baseline HRQL at follow-up Failure to recover associated with septic shock, older age, immune compromise, and LOS
Retrospective cohort study, USA—Czaja et al. (30)	HIC	7,183 children (1 m-18 y) with severe sepsis	*Readmission*—acute hospitalization beyond 2 days from sentinel discharge date *Late mortality*—death occurring > 28 days from discharge date	28-day mortality 6.8% Late mortality 6.5% Readmission 47% Sentinel admission factors associated with adverse outcomes—neurologic, hematological organ dysfunction, government-based insurance, and coexisting health conditions
Prospective, cohort-outcome study, USA—Zimmerman et al. (31)	HIC	389 children (1 m-18 y) with community-acquired septic shock	*Functional status* using PCPC, POPC, and FSS at baseline, day 7 and day 28 or hospital discharge *Parent-proxy assessments of child's HRQL* utilizing the PedsQL, PedsQL Infant Scales, or the Stein-Jessop FSS at baseline, day 7, 1 m, 3 m, and 12 m	At day 28/hospital discharge, 22% exhibited poor gross functional status (POPC ≥ 3 and an increase of ≥ 1 from baseline) total FSS increased to 9.0 (6.0–15.0), median change for PedsQL, comparing baseline and 1 m, was −11.0 (−29.2–5.55) and for FSII-R, was 0.0 (−10.7–10.7) At 1, 3, 6, and 12 m assessments, 39, 32, 27, and 31% of patients assessed with FSII-R remained at least 4.5 points below their baseline HRQL
Prospective, cohort-outcome study, USA—Zimmerman et al. (32)	HIC	259/389 (67%) and 246/389 (63%) of the LAPSE participants could be assessed for outcomes at 1 and 3 m, respectively	*Composite outcome of death or persistent, serious deterioration of HRQL* as compared to baseline—HRQL persisting > 25% below baseline HRQL as assessed at 3 m	Death or PSD-HRQL occurred in 37% and 28% of evaluable patients at 1 and 3 m, respectively Summation of daily PELOD scores, highest VIS, and any acute pathological neurologic sign/event were independently associated with death or PSD-HRQL at 3 m.
Secondary analysis of LAPSE study, USA—Pinto et al. (33)	HIC	117 children (1 m−18 y) from LAPSE study with baseline HRQL score ≤ 80 and survived 3 m and had complete data	*HRQL* using the PedsQL or Stein-Jessop FSS	52% had ≥ 10% improvement in HRQL by 3 m, lower pre-sepsis HRQL was associated with increased odds of improvement at 3 and 12 m, and improvement was more sustained among children without severe developmental delay
Secondary analysis from a prospective inception cohort, Canada, Sidhu et al. (34)	HIC	502 infants ≤ 6 wk admitted after surgery for complex CHD	Full-scale intelligence quotient *(FSIQ)*, the performance intelligence quotient *(PIQ)*, verbal intelligence quotient *(VIQ)*, and adaptive skills assessment	Sepsis occurred in 19% overall and in 19% of survivors By 4.5 years, there were 18% deaths, and 96% had follow-up completed. ECMO was associated with worse scores on all neurocognitive outcomes on multivariable regression and sepsis perioperatively was associated with performance and verbal intelligence quotients.

*LMIC, low- and middle-income countries; HIC, high income country; POPC, Pediatric Overall Performance Category; PCPC, Pediatric Cerebral Performance Category; PICU, pediatric intensive care unit; pSOFA, pediatric sequential organ failure assessment; CPR, cardiopulmonary resuscitation; RESOLVE, REsearching severe Sepsis and Organ dysfunction in children: a gLobal perspective; CNS, central nervous system; PRISM, Pediatric Risk of Mortality; HRQL, Health related Quality of Life; PedsQL, Pediatric Quality of Life Inventory; LAPSE, Life After Pediatric Sepsis Evaluation; FSS, Functional status scale; FSIQ, Full-scale intelligence quotient; PIQ, performance intelligence quotient; VIQ, verbal intelligence quotient; PSD-HRQL, persistent, serious deterioration of HRQL; PELOD, Pediatric Logistic Organ Dysfunction; CHD, congenital heart disease; VIS, vasoactive-inotropic score; ECMO, extracorporeal membrane oxygenation*.

The most common scales used in the pediatric sepsis studies reporting functional outcome were POPC (Pediatric Overall Performance Category), PCPC (Pediatric Cerebral Performance Category), HRQL (Health-related Quality of Life) using PedsQL (Pediatric Quality of Life Inventory), and Functional status scales. Late mortality and readmission were also reported as long-term outcomes. Overall disability reported in PICU survivors ranged between 23 and 50% at hospital discharge or 28 days. Residual disability was measured at various periods of follow-up such as 1, 3, 6, and 12 months from discharge with an overall improving trend with time. Failure to recover from a baseline HRQL from baseline was done at similar time intervals of follow-up, and about one-third of survivors remained 4.5 points below baseline at follow-up. Organ dysfunction scores such as Pediatric Sequential Organ Failure Assessment (pSOFA), Pediatric Logistic Organ Dysfunction (PeLOD), vasoactive inotrope score (VIS), neurological events, immunocompromised status, need for cardiopulmonary resuscitation (CPR), and extracorporeal membrane oxygenation (ECMO) were the common factors associated with poor functional outcome.

In the study by Sankar et al. from India, 121 children aged 2 months to 17 years with severe sepsis were included. Of 82 children who did not have “any disability” at admission in overall function, 39 were found to have “new disability” at PICU discharge (mild = 26, moderate = 11, and severe = 2). Of 36 children with “mild disability” at admission, 6 had “new moderate disability” at PICU discharge. This means that a total of 45 out of 89 children (50.5%) had “new disability” in overall function at PICU discharge. Similarly, a total of 25 out of 89 children (28%) had “new disability” in cognitive function at PICU discharge. At 3 months, 28 (65%) showed improvement in overall function, and 12 (50%) in cognitive function. At 1 year, these numbers increased to 41 (95%) in overall, and 18 (86%) in cognitive function. This means that 5% were left with disability in overall function, and 14% in cognitive function.

In a retrospective cohort study examining data from the RESOLVE (REsearching severe Sepsis and Organ dysfunction in children) trial, Farris et al. included 477 children (term neonates to 17 years old) with severe sepsis and cardiovascular plus respiratory dysfunction. The authors found that 38% of survivors had decline in functional status at 28 days, and 18% had poor functional outcomes.

In another retrospective cohort study by Killien et al., 790 children (1 month to 21 years; term neonates to 17 years old) with sepsis were included. The authors found that 23.8% of survivors failed to recover to their baseline HRQL during follow-up.

In a population-based retrospective cohort study by Czaja et al., 7,183 children (1 month to 18 years) with severe sepsis were included. The authors found that 47% of survivors had emergency readmissions during a median follow-up period of 3 months.

Sidhu et al. carried out a secondary analysis of a prospective cohort study including 97 children with sepsis after congenital heart disease surgery. Of the 76 survivors of sepsis, all had decreased intelligent quotient (IQ) on various scales at 4.5 years.

Zimmerman et al. in their prospective cohort study [LAPSE (Life After Pediatric Sepsis Evaluation) study] included 389 children (1 month to 18 years) with community-acquired septic shock. At 1 month, death or HRQL deterioration was noted in 37%, and the same was 28% at 3 months.

Pinto et al. carried out a secondary analysis of the LAPSE study including 117 children (1 month to 18 years) and found 61 (52%) to have ≥10% improvement in HRQL at 3 months.

While there is scarcity of data on functional outcomes in survivors of pediatric sepsis from LMIC, the overall and cognitive disability rates were comparable to those found in studies from HIC. The important risk factors for poor outcome were also similar. This was based on the authors' assessment of POPC and PCPC scores at admission, discharge, and follow-up. However, most studies in HIC also looked at HRQL with parent questionnaires.

## Discussion

Survivors of pediatric sepsis were reported to have poor functional outcomes in LMIC as well as HIC. POPC, PCPC, and HRQL were the commonly used scales for measuring outcomes. The usual follow-up intervals used to document trend over time were 1, 3, 6, and 12 months from discharge. Common factors associated with poor functional outcome were pSOFA, PeLOD, vasoactive inotrope score, neurological events, immunocompromised status, need for CPR, and ECMO.

### Importance of Measuring Functional Outcomes in LMICs

Despite a huge burden of sepsis in LMIC, there is heterogeneity in patient population and level of care accessible, as well as differences in disease characteristics including type of infections based on the locality. The variation in mortality reported in studies may be contributed by many of these factors. Sepsis-related mortality has reduced overall and may no longer be the sole meaningful outcome. The effects of sepsis extend beyond the hospitalization and include long-term disability, hospital readmission, and late mortality. As survivors from sepsis increase, their long-term outcomes in terms of disability, their need for prolonged care, the QoL of children as well as their caregivers, psychosocial impact, and other such outcomes need to be studied.

A systematic review by Menon et al. found mortality, shock, and organ dysfunction as the commonly reported primary outcome measures in pediatric septic shock trials (35). About one-third of sepsis survivors have mild to moderate disability and poor functional outcome (27). Children with multiorgan dysfunction syndrome (MODS) at PICU admission were more likely to have a discharge POPC score >3 (36.3 vs. 17.4%, *p* < 0.0001) as well as a discharge PCPC score >3 (29.3 vs. 12.2%, *p* < 0.0001), indicating at least moderate impairment in functional status at the time of PICU discharge. Among children discharged in a vegetative or comatose state, 64% had MODS at admission to the PICU (36).

There is limited research on long-term outcomes following critical illness in children. In LMIC, post-discharge mortality may exceed the in-hospital deaths and often go unreported (37). Loss of cases to follow-up adds to this problem. Choosing an appropriate outcome measure is extremely important to assess utility of an intervention in a given setting (38). Core outcome sets designed specifically for LMICs may be needed to overcome the inconsistency in the reported outcomes and improve the quality and relevance for the local region (39, 40).

### Post-ICU Care Syndrome—Pediatrics

PICS is a group of cognitive, physical, and mental health impairments that commonly occur in patients after ICU discharge (41). It may occur as a part of long-term effects of sepsis in PICU survivors (42). Therapeutic interventions like sedation and prolonged mechanical ventilation may contribute to PICS (43). Functional and cognitive impairments may affect growth and development, school performance, and social interactions. However, the child's capacity for growth may be a source of resilience that provides a platform for recovery after critical illness (42).

### Domains of Function

Functional status comprises the ability to perform daily activities of living to meet the basic needs and maintain health (44). It includes mainly the physical, cognitive, psychological, and social function (42).

#### Physical Function

Sepsis through its effects on various organ systems as well as the interventions and medications for organ support may cause various physical impairments. Respiratory dysfunction, difficulty in mobilization, pain, and sensory dysfunction may all lead to inability to take care of oneself (45). ICU-acquired weakness may also contribute to physical limitation due to neuropathy or myopathy. Swallowing difficulty may lead to risk of recurrent aspirations (7). POPC score is widely used to categorize the degree of overall functional impairment (46). Among PICU admissions, physical sequelae were reported in 69% of children, with 30% of them secondary to previous illness and 39% due to acquired morbidity. In 8% of children, the acquired morbidity was related to complications from PICU procedures (47).

#### Cognitive Function

Neurological damage secondary to shock-related ischemia, sepsis-associated inflammation, and metabolic derangements may result in cognitive dysfunction. Prolonged sedation, pain, awareness during ICU stay, delirium, invasive mechanical ventilation, and prolonged length of stay (LOS) in the ICU may also contribute. It may manifest as deficits in memory, attention, and speech. Poor scholastic performance can be seen even in those without obvious cognitive dysfunction. Sepsis increases the prevalence of moderate to severe cognitive dysfunction by almost 10% in relation to non-septic adult patients (48). A Dutch study in children with septic shock reported over 40% cognitive dysfunction in their cohort (49). The PCPC score has been used to estimate global baseline cognitive function and change over the ICU stay (46). These scores may evolve with time with either gradual improvement on follow-up or a few showing further decline (47).

#### Psychosocial Function

Irritability, hyperactivity, anxiety, and emotional and conduct problems are encountered in children discharged following critical illness (50). Psychiatric disorders like depression, hallucinations, delusions, and post-traumatic stress disorder (PTSD) have been reported (51). Higher severity of illness, sepsis, invasive procedures, use of sedative, and paralytic agents have been found to be the risk factors (42). Following discharge, children may have behavioral and social difficulties. Age-appropriate self-report tools have been used for reporting and measuring psychosocial impact on children.

Parents and siblings of children may also experience psychological issues like anxiety, depression, and PTSD. Addressing parental issues is very important as the child and parents form an inseparable dyad and their emotional state has an impact on the child's mental state. Unexpected PICU admissions and more invasive procedures are common risks associated, which are often the case in children with sepsis. In developing countries, parents' level of understanding based on educational status, poverty, and lack of appropriate counseling may add to these issues.

### Health-Related Quality of Life

Health-related quality of life (HRQL) encompasses the impact of health status on physical, mental, emotional, and social functioning. An important measure of health outcomes in children post-critical illness has been proposed (52). Sepsis and many of the treatment modalities used are some of the determinants of poor HRQL, with low socioeconomic status and parental education further contributing to it in developing countries (53). There are over 20 HRQL tools used in pediatric critical care studies. In this review, we have briefly discussed a Pediatric Quality of Life Inventory and a few child health questionnaires. The best tools include both self and proxy reports, cover a wide age range of children, are brief with a low response burden, are multidimensional, and should have internal consistency and test–retest reliability, sensitivity to change over time, and valid content and construct.

### Indicators and Scores

The functional outcomes and HRQL in sepsis survivors largely remain unknown due to lack of valid uniform tools and criteria for assessment in children. Assessment may be time consuming and not practical for large sample sizes. Pediatric functional status assessment tools must also incorporate the rapidly changing norms of growth and development.

#### POPC and PCPC

Fiser developed and validated the POPC and PCPC scales to describe the short-term outcomes after critical illness or injury to quantify overall functional morbidity and cognitive impairment, respectively (46). The POPC includes 6 categories of function (normal, mild, moderate, severe disability, comatose, dead). PCPC includes the same 6 categories with mild, moderate, and severe “cognitive” disability. POPC scoring has been validated against and shown to be associated with measures obtained using the Bayley Psychomotor Developmental Index and the Vineland Adaptive Behavior Scales scores. Stanford–Binet Intelligence Quotients and Bayley Mental Developmental Index scores were significantly different across PCPC categories (54).

#### Functional Status Scale

The Functional Status Scale (FSS) was developed to provide the assessment of functional status suitable for large studies and applicable from full-term newborns to adolescents. It is composed of 6 domains (mental status, sensory, communication, motor function, feeding, respiratory) with domain scores ranging from 1 (normal) to 5 (very severe dysfunction). Total scores range from 6 to 30 with lower scores indicating better function (55). The FSS adds objectivity, increases granularity, and improves quantification of morbidities (54). The Collaborative Pediatric Critical Care Research Network (CPCCRN) conducted a prospective multicentre study in medical and cardiac PICUs assessing functional status at hospital discharge using FSS scores. New morbidity defined as an increase in the FSS of ≥3 was seen in 4.8% with highest among neurological diagnoses (7.3%) followed by acquired cardiovascular disease (5.9%) and cancer (5.3%) (56). New morbidities involved all FSS domains with the highest proportions involving respiratory, motor, and feeding dysfunction.

#### PedsQL 4.0

This scale consists of 4 subscales including physical, emotional, social, and school functioning (57). It is scored from 0 to 100 with physical and psychosocial subscales each scored from 0 to 100. A change of ≥4.5 points between two scores represents the minimum clinically significant difference (29). Conlon et al. studied the long-term HRQL outcomes in children with prolonged PICU stay using the Pediatric Quality of Life Inventory Version 4.0 (PedsQL 4.0) (52).

#### Royal Alexandra Hospital for Children Measure of Function

It is a modification of Children's Global Assessment Scale (CGAS) used in child psychiatry. There are two versions: the Clinical Rating Scale used by clinicians and the Family Rating Scale used by parents and children aged over 10 years (58). The total score of 100 is given with a score between 1 and 30 indicating a poor QoL, 31 and 70 a fair QoL, and a score between 71 and 100 a good QoL. Polic et al. compared QOL in children at 6 and 24 months of PICU discharge using this generic scoring system (59).

#### Questionnaires

For younger children (ages 0–3 years), the Infant–Toddler Quality of Life Questionnaire (ITQOL), Child Health Questionnaire-Parent Form 50 (CHQ-PF50) for 4–18 years filled by parents, and Short Form Health Survey (SF-12) for parents' HRQL have been used (60).

### Studies on Functional Outcomes in Pediatric Sepsis

The European Childhood Life-threatening Infectious Disease Study (EUCLIDS) was a large prospective multicenter cohort study including 795 children admitted with sepsis to 52 European PICUs. The mortality in this study was 6%, and disability at discharge was 31% among the survivors (61).

The studies from LMIC on functional outcome in pediatric sepsis are scarce. In the study by Sankar et al. (26), the common infection sources were respiratory (35%), gastrointestinal (31%), and neurologic (21%) with 37% having comorbidities at admission. The difference in the proportion of children with new disability from the baseline using POPC and PCPC scores was significant (*p* < 0.0001). With increasing duration of follow-up, there was a decrease in the proportion of children with disability with more overall improvement than cognitive.

The analysis of pediatric sepsis survivors of the RESOLVE trial, a multicentric study by Farris et al. (27) for poor functional outcomes, included mostly high-income countries and only one center from middle income countries. POPC scores were used to detect moderate to severe disability at 28 days and no further follow-up for long-term outcomes.

In the HRQL study in survivors of pediatric sepsis by Killien et al. (29), the average parent–child scores of PedsQL 4.0 were obtained for baseline (status during the month prior to admission) and admission and followed up telephonically or electronically 2–12 weeks post-discharge. When controlled for immunocompromised status, the remaining chronic health conditions did not confer excess risk for decline in HRQL. Although illness severity scores did not correlate with poor outcomes, severity of sepsis was associated with failure to recover to baseline.

Czaja et al. (30) looked at the readmission and late mortality among sepsis survivors. Half of the cohort had comorbidities like respiratory disorders, neuromuscular, cardiovascular, and oncologic diseases, and one-fifth were surgical patients. Respiratory infections were the commonest source, and only 15% had multiorgan dysfunction. Late mortality rate was almost equal to 28-day mortality with the highest rate in the first 2 years of discharge. The primary diagnosis for the first readmission was respiratory related, particularly infections, for both with and without chronic illnesses (29 and 36%, respectively). This study shows the long-term effects of sepsis even though functional outcomes were not studied.

### High- vs. Low-Middle Income Countries

Various factors contribute to differences in mortality and morbidity among the high- and middle-income countries. Apart from economic differences, social, and political barriers also play a part (62). Low economic status and lack of education also contribute to poor health seeking behavior. A comparison of outcomes in 2 PICUs with different resources showed higher mortality and poor functional outcomes in the Egyptian center compared to the PICU at Japan (63). There was wide variation in PICU structure in terms of staff number, trained pediatric critical care specialists, and availability of allied healthcare workers. Equipment and utilization of advanced technology was far more in the Tokyo center. The patient characteristics also differed with the PICU in the low-resource setup catering more to medical conditions but with an equal number of post-operative and medical patients in the PICU of HIC. The severity of illness was higher, mortality was more among infants with sepsis and congenital heart disease, and among the survivors, a higher percentage of children had poor cerebral function scores in Egypt. However, this study failed to compare treatment protocols of the 2 units.

### Post-discharge Priorities

Following hospital discharge for sepsis, management should focus on (i) identifying new physical, mental, and cognitive problems and appropriate referral, (ii) reviewing and managing long-term illness and treatment, and (iii) identifying treatable conditions requiring admission. Ongoing evaluation and treatment on an outpatient basis for all survivors of pediatric sepsis for at least the first 3 months after hospital discharge, with particular attention to teenagers, those hospitalized for more than a week, immunocompromised patients, and those with septic shock are recommended (29). In a small subset with chronic illnesses prior to sepsis who experience further deterioration, palliation and symptomatic management may be considered (7).

### Implications in LMIC

The commonly used tools for measuring functional outcome are easily available in LMIC and can be used for monitoring during PICU admission and follow-up. To ensure the application of these, adequate staff training and motivation are the key barriers due to shortage of manpower and high patient load. Training ancillary staff or employing research staff by prioritizing studies on quality improvement could be a first step.

### Recommendations for Improving Functional Outcomes

#### Early Sepsis Care

Favourable functional outcomes could be achieved by early management of sepsis, ICU care and monitoring and rehabilitation (7). Appropriate timely antibiotics, adequate fluid resuscitation, vasoactive support, and source control improve not only mortality but reduce long-term sequelae by decreasing pathogen invasion, better host response, optimizing host–pathogen interaction, and limiting the opportunity for adverse events.

#### Pain, Sedation, and ICU Monitoring

Assessment of pain and requirement of sedation, sedation window when feasible, and monitoring for depth of sedation and delirium would reduce PTSD and cognitive impairment.

#### Mobility and Rehabilitation

Early mobilization can help in reducing physical disabilities and also reduce the duration of ICU stay.

Most of these are low-cost interventions that can be achieved in developing countries. However, hindrances including system-related factors like doctor: patient ratios, training of nurses, improving transport, and triaging remain to be considered.

### The Way Forward

Establishment of good baseline measurements of functional status and QoL is important to associate sepsis and PICU interventions with a change in functional status. A multidisciplinary team approach with the involvement of general pediatricians, physical medicine and rehabilitation, and neuropsychologists is important to identify functional impairments. Identifying risk factors for adverse functional outcomes, early therapy, acute rehabilitation, and prevention of negative impact of various therapies could help in improving functional outcomes. Building clinical infrastructures to include critical illness follow-up clinics may help address the multifaceted needs of sepsis survivors (5). Implementation of a low-cost pediatric sepsis survivorship program is possible by utilizing existing systems of care and can help identify and improve functional outcomes (64). Research in areas to include QoL of sepsis survivors and caregivers needs to be encouraged. Short-term and long-term priorities to enhance sepsis survivorship may be adapted for children from the adult guidelines (5).

## Author Contributions

JS conceptualized the review, searched the literature, helped in writing, and editing the manuscript. NR drafted the manuscript and edited the final version. All authors contributed to the article and approved the submitted version.

## Conflict of Interest

The authors declare that the research was conducted in the absence of any commercial or financial relationships that could be construed as a potential conflict of interest.

## Publisher's Note

All claims expressed in this article are solely those of the authors and do not necessarily represent those of their affiliated organizations, or those of the publisher, the editors and the reviewers. Any product that may be evaluated in this article, or claim that may be made by its manufacturer, is not guaranteed or endorsed by the publisher.
